# Older Adult and Primary Care Practitioner Perspectives on Using, Prescribing, and Deprescribing Opioids for Chronic Pain

**DOI:** 10.1001/jamanetworkopen.2024.1342

**Published:** 2024-03-06

**Authors:** Timothy S. Anderson, Brianna X. Wang, Julia H. Lindenberg, Shoshana J. Herzig, Dylan M. Berens, Mara A. Schonberg

**Affiliations:** 1 Division of General Internal Medicine, University of Pittsburgh, Pittsburgh, Pennsylvania; 2 Center for Pharmaceutical Policy and Prescribing, Department of Medicine, University of Pittsburgh, Pittsburgh, Pennsylvania; 3 Division of General Medicine, Beth Israel Deaconess Medical Center, Boston, Massachusetts; 4 Harvard Medical School, Boston, Massachusetts

## Abstract

**Importance:**

Guidelines recommend deprescribing opioids in older adults due to risk of adverse effects, yet little is known about patient-clinician opioid deprescribing conversations.

**Objective:**

To understand the experiences of older adults and primary care practitioners (PCPs) with using opioids for chronic pain and discussing opioid deprescribing.

**Design, Setting, and Participants:**

This qualitative study conducted semistructured individual qualitative interviews with 18 PCPs and 29 adults 65 years or older prescribed opioids between September 15, 2022, and April 26, 2023, at a Boston-based academic medical center. The PCPs were asked about their experiences prescribing and deprescribing opioids to older adults. Patients were asked about their experiences using and discussing opioid medications with PCPs.

**Main Outcome and Measures:**

Shared and conflicting themes between patients and PCPs regarding perceptions of opioid prescribing and barriers to deprescribing.

**Results:**

In total, 18 PCPs (12 [67%] younger that 50 years; 10 [56%] female; and 14 [78%] based at an academic practice) and 29 patients (mean [SD] age, 72 [5] years; 19 [66%] female) participated. Participants conveyed that conversations between PCPs and patients on opioid use for chronic pain were typically challenging and that conversations regarding opioid risks and deprescribing were uncommon. Three common themes related to experiences with opioids for chronic pain emerged in both patient and PCP interviews: opioids were used as a last resort, opioids were used to improve function and quality of life, and trust was vital in a clinician-patient relationship. Patients and PCPs expressed conflicting views on risks of opioids, with patients focusing on addiction and PCPs focusing on adverse drug events. Both groups felt deprescribing conversations were often unsuccessful but had conflicting views on barriers to successful conversations. Patients felt deprescribing was often unnecessary unless an adverse event occurred, and many patients had prior negative experiences tapering. The PCPs described gaps in knowledge on how to taper, a lack of clinical access to monitor patients during tapering, and concerns about patient resistance.

**Conclusions and Relevance:**

In this qualitative study, PCPs and older adults receiving long-term opioid therapy viewed the use of opioids as a beneficial last resort for treating chronic pain but expressed dissonant views on the risks associated with opioids, which made deprescribing conversations challenging. Interventions, such as conversation aids, are needed to support collaborative discussion about deprescribing opioids.

## Key Points

**Question:**

What are the experiences of older adults and primary care practitioners (PCPs) with using opioids for treatment of chronic pain and discussing opioid deprescribing?

**Findings:**

In this qualitative study analyzing responses to a semistructured interview by 29 older adults prescribed long-term opioids and by 18 PCPs, there was consensus on the use of opioids as a last resort to improve quality of life and that deprescribing conversations were often unsuccessful. There was disagreement on the perceived risks associated with long-term opioid use and the barriers to deprescribing conversations.

**Meaning:**

These findings highlight the need to develop tailored resources to support older adults and PCPs in having successful conversations on the risks and benefits of continued opioid use.

## Teaser

This qualitative study describes the experiences of older adults and primary care practitioners with opioids for treatment of chronic pain and patient-practitioner conversations about opioid deprescribing.

## Introduction

Thirty percent of Americans 65 years or older report chronic pain, and more than 10% report high-impact pain that limits activities.^1^ Pharmacotherapy may result in improved symptoms and function; however, pain medications contribute to polypharmacy and many have a high burden of adverse effects.^2^ In particular, opioid medications are commonly used for treatment of chronic pain but are strongly associated with increased risk for drug interactions, sedation, falls, respiratory depression, addiction, and death due to unintentional overdose.^3^ National awareness of the risks associated with opioid use and the opioid overdose epidemic led to the Centers for Disease Control and Prevention (CDC) developing practice guidelines in 2016 on tapering high-dose opioids or stopping long-term opioids. However, there are concerns that these guidelines have led to adverse outcomes due to overly rapid tapers.^4,5,6,7,8^

In the US, much of the attention on opioids has focused on addiction and harm reduction for younger adults.^4,9^ However, more than 15% of adults 65 years or older filled an opioid prescription in 2019, and 5% received long-term opioids.^10^ Older adult populations face a unique set of risks, with a lower frequency of misuse but heightened risks of medication-related harms related to polypharmacy and multimorbidity. Opioids may also provide substantial benefits to older adults with chronic pain and multimorbidity, particularly promoting physical functioning, which may be vital to achieving other chronic disease goals and emotional well-being.^2,3^ Despite a growing body of literature supporting older adults’ willingness to reduce medications and evidence that collaborative patient-clinician-pharmacist models can effectively reduce the use of high-risk medications, patient and clinician experiences discussing deprescribing opioids are underexplored compared with other drug classes (eg, benzodiazepines).^11,12,13^ Recent qualitative studies from Australia have identified barriers to deprescribing, including lack of alternative treatments, lack of knowledge on how to deprescribe, stigma, and lack of time.^14,15,16,17^

In the US, little is known about patient and clinician views on deprescribing chronic opioids among older adults. Thus, we used qualitative methods to explore older adults’ perspectives and experiences with reducing long-term opioid use and primary care practitioners (PCPs) communication and decision-making around deprescribing long-term opioids. We aim to use this information to develop strategies to support communication between PCPs and older adults around deprescribing opioids.

## Methods

### Setting, Participants, and Study Design

In this qualitative study, we conducted semistructured individual interviews with PCPs and patients at a large Boston-area health system. For each 45-minute phone or video interview, participants provided verbal informed consent, the interview was attended by 1 study physician (T.S.A., J.H.L.) and research assistant (B.X.W., D.M.B.), and participants received a $50 incentive. This study followed the Consolidated Criteria for Reporting Qualitative Research (COREQ) reporting guideline. All interviews took place between September 15, 2022, and April 26, 2023. The study was determined to be exempt human subjects research by the Beth Israel Deaconess Medical Center institutional review board.

#### PCP Participants

The PCP participants were identified from 5 clinics affiliated with the medical center: 1 academic general medicine clinic, 1 academic geriatrics clinic, and 3 community-based primary care clinics. Attending physicians and nurse practitioners were invited to participate via email, and participants completed a brief questionnaire on their demographics and clinical practice.

#### Patient Participants

Patient participants were identified from a pharmacy registry for the academic general medicine clinic, which serves approximately 40 000 patients. Eligible patients were 65 years or older and prescribed long-term opioids, defined as at least 3 prescriptions for 28-day supplies during the prior 180 days. The PCPs were given the opportunity to opt out on behalf of their patients. We sent eligible patients an informational letter that enabled them to opt out of being contacted. Patient participants completed a questionnaire on their demographics, including self-reported race and ethnicity, pain, and medication use. Participants were asked about race and ethnicity given prior research documenting differences in patterns of opioid prescribing to racial and ethnic groups. The questionnaire included the Pain, Enjoyment of Life, and General Activity (PEG) scale, which assesses pain intensity and interference with enjoyment of life and general activity.^18^ To quantify perceived benefits of current opioid use, participants were asked their current PEG scale and their projected PEG scale if they were to stop opioids.

### Interview Guides

Interview guides were developed by the study team, which included 3 PCPs (T.S.A., J.H.L., and M.A.S.) and 2 members (J.H.L., S.J.H.) of the medical center’s opioid care committee based on review of opioid prescribing and broad deprescribing literature. Guides were reviewed with the US Deprescribing Research Network Stakeholder Engagement Council, which is composed of stakeholders who represent patient, family, and various organizational perspectives.^19^ The PCP guide included open-ended questions on experiences caring for older adults with chronic pain, experiences prescribing and deprescribing opioids, and barriers to deprescribing (eTable 1 in Supplement 1). The patient guide included open-ended questions on experiences with chronic pain, experiences with opioids, the perceived benefits and risks of opioids, conversations with clinicians, and experiences tapering opioids (eTable 2 in Supplement 1). Patients were also asked how likely they would be to consider reducing their opioid dose in 9 hypothetical situations, including adverse effects, family member concerns, guideline recommendations, and the development of tolerance.

### Qualitative Analysis

All sessions were audio recorded, deidentified, and transcribed. Field notes were taken by 1 of the study investigators (B.X.W. and D.M.B.). Transcripts were not reviewed by participants. Thematic analysis was conducted using an iterative, multistage, inductive coding process until we reached thematic saturation. The initial codebook was created and refined by 3 investigators (T.S.A., B.X.W., and D.M.B.). Two investigators (B.X.W., D.M.B.) then reviewed the transcripts for accuracy and independently coded the data using NVivo software, version 12; these team members met frequently to clarify themes, and discrepancies were resolved by consensus with a third investigator (T.S.A.). After data were coded, the research team met to discuss the salience of emergent themes. A list of themes, subthemes, and illustrative quotes were produced following a grounded theory approach and were reviewed by the entire research team, including a qualitative research expert (M.A.S.), to produce a final set of themes.^20^ Reported barriers to deprescribing were synthesized using the COM-B (capability, opportunity, motivation, and behavior) model of behavior change, as the act of deprescribing requires new behaviors by both clinicians and patients.^14,21,22^ The COM-B framework has been applied to identify barriers to deprescribing other medications.^23,24^ Direct quotes were used to illustrate themes.

## Results

We contacted 94 PCPs, of whom 21 responded and agreed to participate, and 18 were scheduled. Of the 18 participating PCPs, 12 (67%) were younger than 50 years (10 [56%] were female, 8 [44%] were male, and 5 [28%] self-identified as Asian, 12 [67%] as White, and 2 [11%] as Hispanic). The majority were physicians (17 [94%]), specialized in internal medicine (15 [83%]), and based at an academic practice (14 [78%]) (Table 1).

**Table 1.** Characteristics of 18 Primary Care Practitioner Participants

Characteristic	Participants, No. (%)
Demographics	
Age, y	
30-39	7 (39)
40-49	5 (28)
50-59	4 (22)
≥60	2 (11)
Sex	
Female	10 (56)
Male	8 (44)
Hispanic ethnicity	2 (11)
Race	
Asian	5 (28)
White	12 (67)
≥2	1 (6)
Practice characteristics	
Clinical training	
Physician	17 (94)
Nurse practitioner	1 (6)
Practice location	
Academic hospital–based	14 (78)
Community-based	4 (22)
Time in practice, y	
<5	9 (50)
5-9	3 (17)
≥10	6 (33)
Patient panel size	
<500	7 (39)
500-1000	5 (28)
>1000	6 (33)
Specialty	
Internal Medicine	15 (83)
Infectious Disease	1 (6)
Geriatrics	1 (6)
Addiction	1 (6)

Of 82 eligible patients, 31 agreed to participate and 29 were scheduled (eFigure in Supplement 1). The 29 participating patients had a mean (SD) age of 72 (5) years, 19 (66%) were female, 10 (34%) were male, and 10 (34%) self-identified as Black and 19 (66%) as White. Most participants reported experiencing chronic pain for more than 5 years (23 [80%]) and using opioids for more than 5 years (18 [62%]). Most participants used low opioid doses (median [IQR] daily oral morphine milligram equivalents, 21.3 [10.9-41.3]; range 5.0 to 240.0). Patients reported high daily PEG scores (mean [SD], 6.6 [2.3]) and predicted worsening without opioids (mean [SD] anticipated increase in PEG score, 1.4 [1.7]) (Table 2).

**Table 2.** Characteristics of 29 Patient Participants

Characteristic	Patients, No. (%)
Demographics	
Age, mean (SD), y	72 (5)
Sex	
Female	19 (66)
Male	10 (34)
Hispanic ethnicity	1 (3)
Race	
Black	10 (34)
White	19 (66)
Chronic pain experience	
Primary pain syndrome	
Back or neck	26 (90)
Headache	4 (14)
Muscle	14 (48)
Joint	23 (79)
Nerve	20 (69)
Cancer-related	5 (17)
Other	9 (31)
Duration of pain, y	
<2	1 (3)
2-5	5 (17)
6-10	10 (35)
>10	13 (45)
Nonopioid medication	
Acetaminophen	18 (62)
Nonsteroidal anti-inflammatory	8 (28)
Muscle relaxant	8 (28)
Neuropathic medication	9 (31)
Other	5 (17)
Opioid medication	
Tramadol	9 (31)
Oxycodone	15 (48)
Other	7 (24)
Duration of opioid therapy, y	
<2	5 (17)
2-5	8 (27)
>5	18 (62)
Daily opioid dose, median (IQR) [range], MME	21.3 (10.9-41.3) [5.0-240.0]
PEG score^a^	
Current PEG score, mean (SD)	
Pain intensity	6.8 (2.4)
Interference with enjoyment of life	6.3 (2.9)
Interference with general activity	6.5 (2.6)
Overall	6.6 (2.3)
Anticipated increase in PEG score without opioid use, mean (SD)	
Pain intensity	1.1 (1.7)
Interference with enjoyment of life	1.9 (2.9)
Interference with general activity	1.6 (2.2)
Overall	1.4 (1.7)

Abbreviations: MME, morphine milligram equivalent; PEG, Pain, Enjoyment of Life, and General Activity.

^a^ PEG score scaled from 0 to 10 for each category, with higher scores representing greater intensity or interference.

All participants conveyed that conversations between clinicians and patients on opioid use and chronic pain were frequently challenging and that conversations regarding medication risks and deprescribing were uncommon. While patients and PCPs broadly shared goals related to opioid use, each group highlighted different risks of opioids and barriers to opioid deprescribing. Major themes were structured into 3 categories: experiences with chronic pain and opioid management; experiences with deprescribing; and barriers to deprescribing conversations.

### Experiences With Chronic Pain and Opioid Management

#### Shared Themes

Both groups reported exhausting alternatives before using opioids, and patients reported using many adjunctive treatments alongside opioids (Table 3). Most PCPs expressed that they start opioids as a last option when other options are ineffective, not tolerated, or contraindicated due to comorbidities or drug interactions. Patients reported a wide range of treatments, including over the counter pain relievers, physical therapy, meditation, counseling, other prescription medications, and clinician-administered injections. There was consensus that opioids can help older adults achieve their functional goals and improve quality of life. The PCPs focused on “balancing quality of life with the risk of adverse effects” (PCP identifier, PCP18). Patients agreed that opioids rarely entirely rid them of pain but often helped increase their ability to function.

**Table 3.** Patient and PCP Experiences With Chronic Pain, Opioid Use, and Deprescribing

Theme	Explanation	Quotations
Shared themes		
Opioids used as a last resort	PCPs and patients use other modalities to treat pain before using opioids.	PCP: “I only reserve [opioids] for patients who have failed other analgesics or are allergic to NSAIDs or they have contraindications, or they have polypharmacy and bad interactions” (PCP16).
		Patient: “So I will take Tylenol, the arthritis strength Tylenol, sometimes that helps and I relax, you know? When that doesn’t help, I will take a tramadol and that might help” (PT28).
Functional goals and quality of life	PCPs and patients agree that opioids can improve functionality and quality of life.	Patient: “When I take the pill, it’s like I’m able to do anything and everything. Sometimes if I don’t take the pill, walking the dog, it’s a chore” (PT15).
		PCP: “I think more of the conversation tends to be focused on functionality and how we can get them to live a life that feels like it enables them to still have the same, basically the same, level of activity that they desire” (PCP1).
Patient and clinician trust	PCPs and patients agree that trust is fundamental in having successful conversations about opioids.	PCP: “Nothing is going to work in terms of caring for these folks or any patients until you build their trust” (PCP10).
		Patient: “My doctor knew my history, so she understood that I wasn’t seeking drugs just to become a drug addict. She realized that I was really in pain” (PT15).
Lack of success	PCPs and patients report low success with deprescribing opioids.	PCP: “I haven’t [tapered] very successfully or stopped it very often . . . and it felt like a failure” (PCP9.
		Patient: “My doctor has always kind of brought it up. . . . But I tell him there’s no problem. I don’t feel that I have any problems as far as the medication is concerned” (PT25).
Conflicting themes		
Perceived risks of opioids	PCPs and patients have differing perceptions on the risks of opioids.	PCP: “The negative impacts of those medications can be considerably more significant as you age in terms of altered mental status and fall risks” (PCP14).
		Patient: “I don’t want to get into a cycle of taking the Percocet and then getting addicted” (PT22).
Initiation of deprescribing conversations	PCPs and patients report differences in who initiates opioid deprescribing conversations.	Patient: “As far as the written prescription, it always stays the same, but like I said before, a lot of times I don’t take 4 Vicodin a day on a regular basis. I usually take 2 or 3” (PT25).
		PCP: “Most of the time it’s brought up by me . . . not from the patient” (PCP12).

Abbreviations: NSAID, nonsteroidal anti-inflammatory drug; PCP, primary care practitioner; PT, patient.

Both groups reported that having an established trusting relationship between clinician and patient is important to having conversations about long-term opioid medications. The PCPs reported difficulties when inheriting patients who initiated long-term opioids through other practitioners: “It is the patients that I’ve inherited that have been the most challenging because there have been other expectations from other providers” (PCP5). Similarly, patients reported feeling comfortable discussing opioids with long-term clinicians, but challenges most often arose with new clinicians or those providing episodic care.

#### Conflicting Themes

The PCPs and older adult patients had differing perceptions on the risks of opioids. The PCPs were primarily concerned about opioid-related adverse drug events associated with aging, particularly falls and confusion. Patients were primarily concerned with the risk of addiction, infrequently identified other risks associated with opioids, and reported rarely having conversations with clinicians about risks other than addiction. Some patients were aware of constipation as an adverse effect and medication interactions, but no patient reported awareness of increased risks of confusion, falls, fatigue, or difficulty breathing. Multiple patients brought up the use of contractual agreements as a time when there were conversations on risks. For example, patient identifier 18 (PT18) indicated, “[My doctor] kind of skimmed over them when I was presented with the contract. Since I signed the contract, [my doctor] hasn’t even mentioned it again.”

### Experiences Deprescribing

The PCPs reported typically initiating deprescribing conversations after an adverse drug event occurred. The PCPs noted that deprescribing takes time and requires open conversations with patients and that patients rarely initiate deprescribing conversations. One PCP (PCP8) stated that “It’s better to ally with the patients and not set really clear expectations up front and then go slow.” Patients reported that deprescribing was less often initiated by clinicians and more often by their own decision to take fewer opioids than prescribed or to ask for a less strong medication.

While 16 patients reported having a prior conversation about deprescribing with their PCP, only 6 patients reported a prior taper attempt, and 4 successfully tapered to a lower dose. Of the 10 patients who discussed deprescribing but did not taper, reported reasons included worries of increased pain and adverse effects and a lack of any problems to prompt a taper. Both patients and PCPs believed that structured opioid tapering was often unsuccessful. One PCP (PCP18) stated, “Even if patients are willing to trial a lower dose . . . they feel like things are not as good as their usual dose and would ask to go back.”

### Barriers to Deprescribing

**Figure 1.**
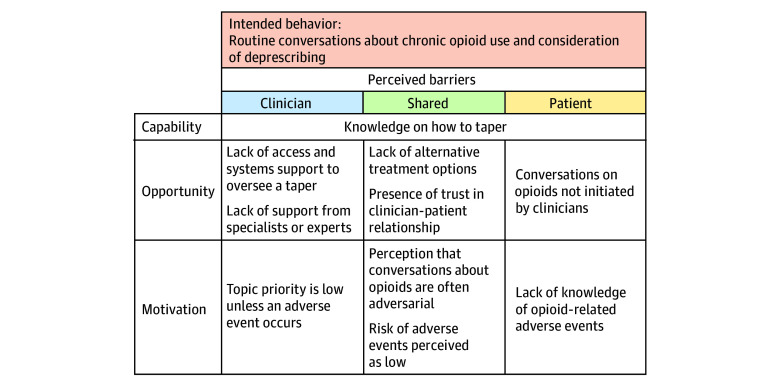
Conceptual Model of Clinician and Patient Perceived Barriers to Opioid Deprescribing

Barriers to opioid deprescribing were grouped using the COM-B model framework (Figure 1) and are detailed below. The PCPs also identified a range of potential ideas for supporting opioid deprescribing, including risk stratification tools, prescribing guidelines targeting older adults, and educational handouts (eTable 3 in Supplement 1).

#### Capability to Deprescribe

Both groups identified lack of knowledge on opioid deprescribing as a barrier. Several PCPs described gaps in knowledge on how to safely taper opioids. Some PCPs described helpful team-based care with support from pharmacists or addiction medicine specialists, while others expressed frustration that specialist referrals were rarely helpful.

#### Opportunity to Deprescribe

Another common barrier was “the lack of pharmaceutical options that are safer alternatives” (PCP12). Both groups noted this barrier was greater for older adults due to contraindications to other treatments related to having multiple chronic conditions. A second barrier noted by PCPs was the lack of time to have deprescribing conversations and to begin a taper. Multiple PCPs highlighted barriers in the appointment access and system supports needed to manage a taper, stating, “I can’t see patients on a weekly basis, so that either means we don’t do it, or we do a suboptimal job” (PCP9).

The PCPs reported that conversations about opioids occurred regularly but rarely led to deprescribing due to barriers such as patient resistance, lack of dedicated time, and lack of alternatives. Patients, on the other hand, reported that clinicians rarely initiated conversations about long-term opioids or deprescribing them. One patient (PT25) commented, “In the last 2 years he has never brought it up; I have never brought it up. We just keep doing what we’re doing because it seems to work.”

#### Motivation to Deprescribe

Several PCPs reported patient resistance to deprescribing opioids, describing patients as defensive, adversarial, attached, and scared to stop opioids. The PCPs expressed that some patients had previous negative experiences that led to hesitation around tapering. “There is the fear of withdrawal because some people have been tapered in a way that was aggressive in the past and felt terrible” (PCP14). Patients reported not being educated by clinicians about opioid risks. One patient (PT3) said that “they assumed I knew any other risks,” whereas another patient (PT27) stated that “nobody actually talked to me about the risks of taking these medications.”

The majority of patients reported having at least 1 negative experience related to opioids with a clinician, generally not their PCP. Negative interactions stemmed from either not feeling heard by a clinician or being stigmatized for taking opioids, feeling that a doctor “was labeling me as someone who is a drug abuser” (PT23). In contrast, conversations with PCPs were often described as positive: “They understood my apprehensions about [tapering]. Just in case this doesn’t work, we’ll still be there and we will be able to go back to former dosages in the event that I was unsuccessful (PT1)”.

The PCPs reported viewing deprescribing as important but lower priority due to competing tasks. One PCP (PCP17) stated that “There’s a lot to address in a typical session, and these conversations can be difficult and time consuming.” Patients viewed deprescribing as low priority unless they had a problem with their medication, with 1 patient (PT24) stating, “If there was a problem, I’d have no problem talking to [my PCP] about it. But there never has been.”

### Differences in Themes Between Patients With Lower vs Higher PEG Scores

When comparing responses between patients with lower vs higher PEG scores, 3 differences emerged (eTable 4 in Supplement 1). Patients with lower PEG scores expressed more awareness of opioid risks beyond addiction and shared more negative past experiences with clinicians. Patients with higher PEG scores were broadly less open to deprescribing opioids.

### Patient Likelihood to Consider Deprescribing

**Figure 2.**
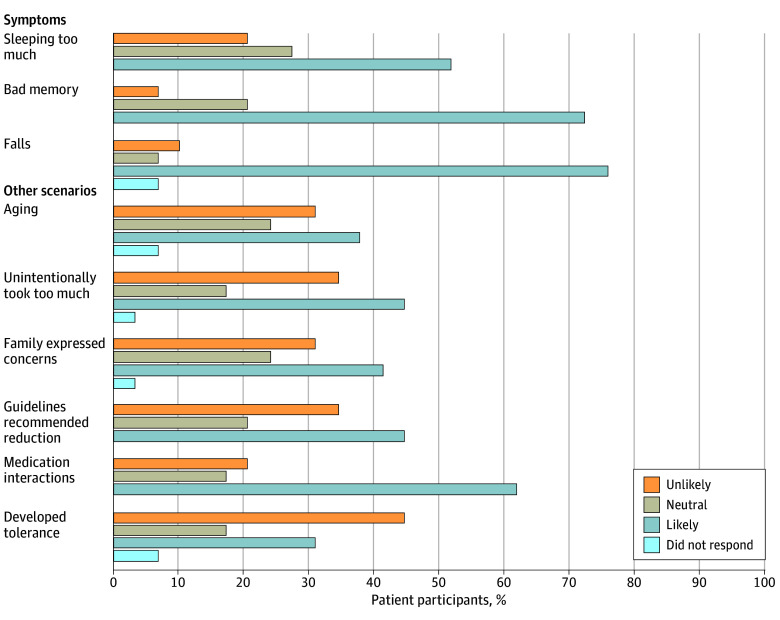
Patient Willingness to Consider Opioid Deprescribing Given Hypothetical Scenarios

Figure 2 demonstrates that when prompted with hypothetical scenarios, patients were most likely to consider deprescribing when experiencing adverse effects of opioids, such as falls, impaired memory, and sedation, or if needing to take medications that interacted with opioids. Participants were less enthusiastic about deprescribing when scenarios focused on concerns about aging, family member concerns, guideline or expert recommendations, or unintentionally taking extra opioids. Patients were unlikely to consider deprescribing when the scenario focused on developing tolerance to opioid, in which case multiple patients indicated they would require more opioids.

## Discussion

Findings of this qualitative study indicated that the use of opioids for treatment of chronic pain was viewed as a challenging topic by older adults and PCPs. Both groups aimed to minimize use due to concerns for harms and noted that opioids can be a beneficial last resort but expressed differing views on the risks of opioids. While older adults focused on concerns of addiction, they were largely unaware of physical dependence or adverse drug events associated with opioids. The PCPs were less concerned about misuse among older adults but were primarily concerned about adverse drug events, such as falls and confusion. Communication around opioid risks and deprescribing was viewed as challenging and infrequent by both groups. These results highlight potential targets for developing tailored resources, such as conversation aids, to support older adults and PCPs in having successful conversations on the risks and benefits of continued opioid use.

Our findings are consistent with recent Australian qualitative studies that identified similar barriers to deprescribing and emphasized the importance of trust.^14,15,16,17^ In addition, a recent US survey of PCPs in 1 health system identified that nearly half of them lacked confidence in developing opioid tapering protocols.^25^ A recent Veterans Administration study of adults of all ages identified discordance between clinicians and patients in the goals of long-term opioid therapy.^26^ By exploring the unique situations faced by older adults with chronic pain and their PCPs in the US, our study expands on prior work in several important ways.

Our findings highlight the barriers facing successful implementation of national guidelines on opioid prescribing and the importance of contextualizing conversations on opioid use for older adults. Widespread awareness of the opioid epidemic has led prescription opioid use to drop precipitously in the US.^8,27^ Despite revised 2022 CDC guidelines recommending discussion of the benefits and harms of long-term opioid therapy every 3 months,^28^ most patient participants in our study reported that conversations about opioid use were rare, with reasons including perceived discord when discussing opioids, other health conditions being higher priority, and a lack of alternatives for pain management. Participants were in agreement with CDC guidance that older adults face particular challenges in managing chronic pain due to increased risks of both opioid and alternative medications and the importance of prioritizing function.

Despite the importance of safely managing chronic pain and opioid prescribing, PCPs identified a lack of resources to inform conversations about choices for chronic pain management and making decisions on long-term opioid use. Research developing conversation aids are urgently needed to help guide decisions on chronic pain and long-term opioid therapy for older adults.^29,30^ In Australia and the Netherlands, new educational materials, including opioid deprescribing practice guidelines targeting clinicians^31,32^ and materials targeting older adults,^33^ are being developed. If those interventions are successful, efforts to study the feasibility of implementing and tailoring them to US populations will be needed.

Our findings indicate that barriers related to time are likely to require health system changes and investment. The CDC guideline^28^ advises following up at least monthly when patients are tapering opioids, a suggestion which PCPs in our study agreed with but identified as impractical. Pharmacist-led models have demonstrated promise for other deprescribing interventions, but these models may be more challenging for opioids given the importance placed on existing relationships. In a promising development, in 2023, Medicare released new monthly bundled payment codes for managing chronic pain,^34^ which has the potential to spur the greater time spent on developing a long-term care relationship needed to support safe and patient-centered pain management, including conversations on opioid prescribing and deprescribing.

### Limitations

There are limitations to our study. The study took place in a single academic medical center, and the results may not be generalizable to other settings (eg, rural, private practice, or other regions). While we attempted to capture a diversity of perspectives, our study was not designed to compare subgroups.

## Conclusions

The findings of this qualitative study suggested that PCPs and older adults viewed opioid use as an effective last resort for treating chronic pain and expressed discordant views on the risks of opioids and reasons opioid deprescribing is often unsuccessful. Safely reducing opioid use among older adults with chronic pain is likely to require the development of materials to foster more informed conversations on the benefits and harms of opioids as well as payment and policy interventions to support the time and teams needed for deprescribing opioids.

## Notes

Supplement 1. **eTable 1.** Primary Care Practitioner Participant Interview Guide **eTable 2.** Patient Participant Interview Guide **eFigure.** Patient Participant Flowchart **eTable 3.** Suggestions from Primary Care Practitioners on Tools and Resources to Support Conversations on Opioid Prescribing and Deprescribing **eTable 4.** Differences in Patient Experiences With Chronic Pain, Opioid Use, and Deprescribing by Pain, Enjoyment of Life, and General Activity Scale Scores

Supplement 2.
Data Sharing Statement
