# Supplementary material for: Older Adult and Primary Care Practitioner Perspectives on Using, Prescribing, and Deprescribing Opioids for Chronic Pain
Source: JAMA Netw Open. 2024 Mar 6;7(3):e241342. doi: 10.1001/jamanetworkopen.2024.1342 (PMC10918495; doi:10.1001/jamanetworkopen.2024.1342)
Supplement: Supplement 1. — eTable 1. Primary Care Practitioner Participant Interview Guide eTable 2. Patient Participant Interview Guide eFigure. Patient Participant Flowchart eTable 3. Suggestions from Primary Care Practitioners on Tools and Resources to Support Conversations on Opioid Prescribing and Deprescribing eTable 4. Differences in Patient Experiences With Chronic Pain, Opioid Use, and Deprescribing by Pain, Enjoyment of Life, and General Activity Scale Scores [file jamanetwopen-e241342-s001.pdf]

Supplementary Online Content

Anderson TS, Wang BX, Lindenberg JH, Herzig SJ, Berens DM, Schonberg MA. Older adults' and primary care practitioners' perspectives on using, prescribing, and deprescribing opioids for chronic pain. *JAMA Netw Open*. 2024;7(3):e241342. doi:10.1001/jamanetworkopen.2024.1342

eTable 1. Primary Care Practitioner Participant Interview Guide
eTable 2. Patient Participant Interview Guide
eFigure. Patient Participant Flowchart
eTable 3. Suggestions from Primary Care Practitioners on Tools and Resources to Support Conversations on Opioid Prescribing and Deprescribing
eTable 4. Differences in Patient Experiences With Chronic Pain, Opioid Use, and Deprescribing by Pain, Enjoyment of Life, and General Activity Scale Scores

This supplementary material has been provided by the authors to give readers additional information about their work.

eTable 1. Primary Care Practitioner Participant Interview Guide

1. Please describe your experiences caring for older adults with chronic pain.
   a) What about your experiences caring for older adults who use opioids for chronic pain?

2. What role do opioids have in the management of older adults with chronic pain?

3. What is your comfort level in prescribing opioids for older adults with chronic pain?
   a) Why do you feel this way?

4. How does a patient's age affect your use of opioids for chronic disease management?

5. Please describe any experiences in helping older adults taper or stop chronic opioids?
   a) How did this come up?
   b) How did the conversation make you feel?
   c) How did it go?
   d) What made/makes these conversations/situations hard?
   e) What made/makes the conversation go well?

6. Please describe any barriers you have faced in helping older adults taper or stop chronic opioids?

7. What tools do you use, if any, to help you with these conversations?
   a) Are there any local tools in the hospital or health system? Are there national tools or resources?
   b) What tools or supports would be helpful for talking to older adults about tapering or stopping opioids?

8. How does the type of opioid affect your thinking about tapering or deprescribing opioids?
   a) For example, tramadol compared to oxycodone, long acting compared to short acting opioids.

9. Anything else I should have asked you but did not? Any additional thoughts?

eTable 2. Patient Participant Interview Guide

1. Please describe your experiences with chronic pain.
    a) How does chronic pain impact your daily life?

2. Please describe your experiences using medications to help manage your chronic pain.
    a) Please describe the benefits you've experienced from using these medications.
    b) Please describe any side effects or problems you've experienced from these medications.
    c) Please describe any impact the medications have on your quality of life.

3. What kind of conversations do you have with you doctors about your opioid medications?

4. Please describe any conversations you have had with your doctors about the possible downsides or risks of the opioid pain medications you take.

5. Please describe any experiences you've had in decreasing the dose of the chronic opioid?
    a) How did this come up?
    b) How did the conversation make you feel?
    c) If you made a change, how did it go?
    d) What makes these conversations/situations hard?
    e) What made the conversation go well?

6. What sorts of problems would make you want your doctor to talk to you about decreasing the dose or stopping your opioids? *Initially ask open-ended*.

7. I'll now mention a few different issues that sometimes make patients and doctors consider decreasing the dose or stopping your opioid pain medications? For each issue, please rank whether if the issue would make you consider reducing the dose of your pain medication. We will use a 1 to 5 scale, with 1 being very unlikely to reduce medication dose, 3 being neutral and 5 being very likely to reduce medication dose
    a) Sleeping too much / feeling groggy all day
    b) Worsening memory
    c) Falls
    d) Aging increasing your risk of problems
    e) Accidentally took too much
    f) Family expressed concerns
    g) Guidelines say you are taking too much
    h) Taking other medications that can interfere with opioids
    i) Feeling like your current dose was no longer giving you the same pain relief

8. If a doctor asked you to decrease the dose of your opioid pain medications, what questions would you have? What concerns would you have? *Initially ask open-ended questions.*
    a) Probing questions: withdrawal, more pain, support etc?

9. Have you ever had any negative interactions with health care providers related to your chronic pain or use of pain medications? *Specifically, stigma or judgment?*

10. Have you received any helpful resources about the benefits and risks of opioids?

11. Anything else I should have asked you but did not? Any additional thoughts?

eFigure. Patient Participant Flowchart

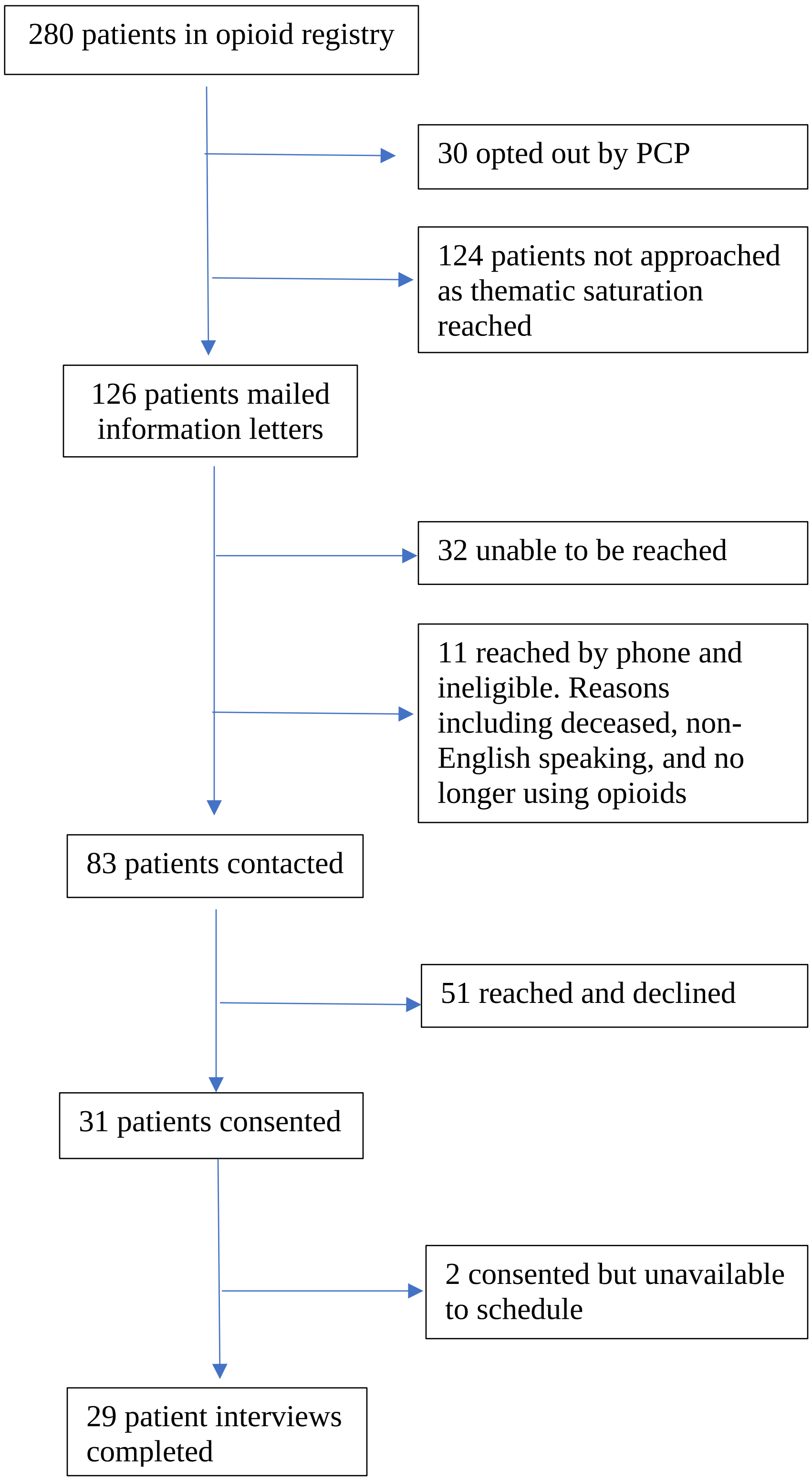

eTable 3. Suggestions from Primary Care Practitioners on Tools and Resources to Support Conversations on Opioid Prescribing and Deprescribing

**Tool**	**Quotes**
Targeted guideline	I wish there was a national guideline for how to approach opioid prescribing and prescribing in adults over 75.(PCP 15)
Risk stratification	A risk stratification tool the same way we have a breast cancer risk population tool that can tell us is this particular patient is a low, average, intermediate, or high risk for short acting opioids versus the patch versus something else. (PCP 13)<br><br>Perhaps the same way that we can equate patients with hemoglobin A1c greater than nine, targeting patients who are either over 65, and or on more than 100 mg of morphine equivalent. And so targeting those folks who are sort of the highest risk. And thinking of sort of a real framework and process to help support those patients. (PCP 3)
Handouts	I think handouts with just something like this class of medications has these lists of 10 problems... And that has alternative approaches. I get a lot of questions about chiropractor treatment, acupuncture, cupping, so alternative medicate, alternative treatment, holistic treatment. (PCP 13)<br><br>A one pager on frameworks or resources that we have would be helpful. Think anything more than that would be too much, too much to read and too much to look at. (PCP 6)
Consults	A virtual consulting mechanism would be brilliant. (PCP 17)
Population Health	I would want us to approach older adults on chronic opiates in the same population health framework that we approach diabetes and hypertension, where we have a registry, where we have a group of people who are making sure that we're sort of checking the boxes we're supposed to check around patients who are on them. (PCP 3)

eTable 4. Differences in Patient Experiences With Chronic Pain, Opioid Use, and Deprescribing by Pain, Enjoyment of Life, and General Activity Scale Scores

Theme	Explanation	Quotes
Patient and Clinician Trust	Patients with lower PEG scores reported prior negative experiences with clinicians while patients with higher PEG scores reported positive experiences with their PCP.	**Low PEG:** I'm not some drug addict off the street, you know what I mean? And they are treating me like I was some guy off the street who was hooked on oxycodone (PT 165).<br><br>**High PEG:** I have a very good relationship with my doctor and she has been my doctor, probably twenty five years and I never had any issues with her (PT164).
Perceived Risk of Opioid	Patients with lower PEG scores were more often aware of risks of adverse drug effects from opioids while patients with higher PEG scores were more often concerned with risk of addiction.	**Low PEG:** If I had side effects which I perceived as being attributable to opioids or if I had some untoward effects that come from opioids that would be the only thing. It would be falling down; respiratory depression (PT217).<br><br>**High PEG:** I'm so afraid of getting dependent on the opioids or addicted to the medication. Almost every time that we have an appointment, you know, I'm talking about the medications and how can I back off some of this (PT49).
Openness to Deprescribing	Patients with higher PEG scores generally reported being less open to opioid deprescribing. This theme was not seen in patients with lower PEG scores.	**High PEG:** If it gave me some kind of problem I probably would, but like it works for me. But I've never felt like I need to decrease it or anything like that. (PT74)

Note: PEG, Pain, Enjoyment of Life, and General Activity Scale, scaled from 0 to 30. Low PEG score group defined as PEG≤10 (N=4). High PEG score group defined as PEG>20 (N=8)
