# Supplementary material for: Older Adult and Primary Care Practitioner Perspectives on Using, Prescribing, and Deprescribing Opioids for Chronic Pain
Source: JAMA Netw Open. 2024 Mar 6;7(3):e241342. doi: 10.1001/jamanetworkopen.2024.1342 (PMC10918495; doi:10.1001/jamanetworkopen.2024.1342)
Supplement: Supplement 2. — Data Sharing Statement [file jamanetwopen-e241342-s002.pdf]

Data Sharing Statement

Anderson. Older Adult and Primary Care Practitioner Perspectives on Using, Prescribing, and Deprescribing Opioids for Chronic Pain. *JAMA Netw Open*. Published March 06, 2024. doi:10.1001/jamanetworkopen.2024.1342

Data
Data available: No

Additional Information
Explanation for why data not available: Codebooks are available as part of the supplement.
